# Feeding Practices and Early Childhood Caries: A Cross-Sectional Study of Preschool Children in Kanpur District, India

**DOI:** 10.1155/2013/275193

**Published:** 2013-12-05

**Authors:** Santhebachalli Prakasha Shrutha, Grandim Balarama Gupta Vinit, Kolli Yada Giri, Sarwar Alam

**Affiliations:** ^1^Department of Pedodontics & Preventive Dentistry, Rama Dental College & Hospital, Kanpur, Uttar Pradesh 208024, India; ^2^Department of Oral & Maxillofacial Surgery, Rama Dental College & Hospital, Kanpur, Uttar Pradesh 208024, India; ^3^Department of Oral & Maxillofacial Surgery, Institute of Dental Sciences, Bareilly, Uttar Pradesh 243006, India; ^4^Staff Residence, Rohilkhand Medical College and Hospital Campus, 19 Red Building, Pilibhit Bypass, Bareilly, Uttar Pradesh 243006, India

## Abstract

*Background*. Early childhood caries (ECC) is a public health problem due to its impact on children's health, development, and wellbeing. The objective of this study was to assess the caries experience in 3–5-year-old children and to evaluate the relationship with their mothers' practices regarding feeding and oral hygiene habits in Kanpur. *Method*. A cross-sectional survey was undertaken on 2000 (974 boys and 1026 girls) children aged 3–5 years from a random sample of preschools in Kanpur district, India. Dental caries experience was recorded using WHO criteria. A pretested questionnaire with 9 questions was used for collecting information regarding mothers' practices regarding feeding and oral hygiene practices. Chi-square test (*χ* ^2^) and Student's *t*-test were used for statistical analysis. *Results*. The prevalence of ECC was 48% with mean dmft of 2.03 ± 2.99. Boys (57%) were affected more than girls (43%) which was found to be statistically significant (*P* < 0.05). Caries prevalence was high and statistically significant (*P* < 0.05) among those who were breast fed for longer duration, during nighttime, those falling asleep with bottle, and those fed with additional sugar in milk. *Conclusion*. Determining the role of feeding practices on early childhood caries can help in the development of appropriate oral health promotion strategies.

## 1. Introduction

Dental caries is still a major oral health problem in most industrialized countries, affecting 60–90% of schoolchildren and the vast majority of adults. It is also a most prevalent oral disease in several Asian and Latin-American countries, while it appears to be less common and less severe in most African countries [1]. Within-country disparities are also common, with preschool children from disadvantaged communities generally experiencing higher levels of disease than the general population [2, 3].

Despite credible scientific advances and the fact that caries is preventable, dental decay in the primary dentition of young children continues to pose a serious threat to child welfare. Early childhood caries (ECC) has been defined as “the presence of one or more decayed, missing due to caries, or filled tooth surfaces in any primary teeth in children under 6 years of age” [4, 5]. Due to its high prevalence, impact on quality of life, potential for increasing risk of caries in the permanent dentition, and role in oral health inequalities, ECC is recognised as a serious public health problem [3]. Socioeconomic, sociocultural, and sociobehavioural determinants are believed to influence specific risk factors for ECC such as dietary and feeding practices, oral hygiene, and dental attendance patterns [6, 7].

In developing countries like India, changing lifestyle and dietary patterns are markedly increasing the caries incidence [8]. Mothers are primary promoters of oral hygiene and they have a major influence on the dietary habits and food choices of children. Patterns of behaviour learnt in early childhood are deeply ingrained and resistant to change. Mothers have an important role in this aspect [9]. Significantly, more mothers of children with caries lack knowledge about some of the determinants and prevention of caries. It is assumed that an increase in the knowledge of mothers will influence their self-care habits and dietary practice and, in turn, improve the dietary and oral hygiene habits of children to prevention of caries [10].

Information on caries prevalence and severity forms the basis for the magnitude and quality of caries prevention programs and treatment needs in a population [11]. There is a paucity of data on ECC in the Kanpur city of India. Therefore, this study was aimed at assessing the caries experiences in 3–5-year-old children and evaluating the relationship with their mothers' practices regarding feeding and oral hygiene habits in Kanpur.

## 2. Materials and Method

A cross-sectional study was conducted among 2000 (974 boys and 1026 girls) preschool children aged 3–5 years of Kanpur city, India from October 2012 to February 2013. Kanpur is located on the banks of the river Ganges and is an important industrial centre. The study protocol was reviewed by the Institutional Review Board and was granted ethical clearance. An approval for this project was obtained from the concerned authorities of Education Department. A written informed consent was obtained from the parents of all children who were willing to participate in the survey.

### 2.1. Training and Calibration

Calibration procedures were performed in the Department of Pedodontics and Preventive Dentistry prior to and during the study to ensure that a consistent standard of the diagnosis was maintained. Reexaminations were carried out on approximately one in ten children selected at random to have a constant check on the intraexaminer variability (kappa value = 90%).

### 2.2. Pilot Survey

A pilot study was carried out among 50 children from one private and one public school to determine the feasibility of the study. Depending on the prevalence obtained for dental caries (44%), 95% confidence level, and 5% allowable error, the sample size was determined to be 1971 which was rounded off to 2000.

### 2.3. Sampling Technique

The sample included children of specified age groups attending various play homes/preschools in Kanpur District of Uttar Pradesh. Preschools are generally situated in urban centres, however, children attending these preschools are drawn from across the district catchment areas, which includes children living in rural home addresses.

Study sample was recruited by a two-stage cluster sampling technique. For study purpose, Kanpur district was arbitrarily divided into 4 geographical regions and schools from each region were randomly selected to obtain the desired sample size, such that there was an equal representation from each of the four zones. Out of the total number of public (39) and private schools (77), fifteen public and thirty five private schools were randomly selected. In the second stage, eligible schoolchildren were stratified, according to gender, and randomly selected in proportion to the total number of students enrolled in each school to reach the sample of around 2000.

### 2.4. Methodology

All children aged between 3 and 5 years attending the selected schools, accompanied by their mothers on the parent teacher meeting day, were invited to participate. A pretested questionnaire consisting of 9 questions, designed in local language (Hindi), was used for collecting all the required and relevant information regarding personal data, mothers' practices regarding feeding, and oral hygiene practices. The questions had 3–5 alternatives and mothers were asked to tick one among them. Children of those parents who gave the informed consent and duly filled questionnaire were clinically examined for dental caries using dentition status and treatment need as per World Health Organisation (WHO) criteria (1997) [12]. Specific codes from the dentition status were subsequently used for calculation of the “dmft” index score, to indicate caries experience. For the (d) component, this included dentition status: “Decayed” and “Filled with decay,” for the (f) component: “Filled, no decay,” and for (m) component: “Missing as a result of caries.”

### 2.5. Statistical Analysis

The recorded data were analyzed using Statistical Package for Social Sciences version 15 software (SPSS Inc., Chicago, IL, USA). Descriptive statistics included computation of percentages, means, and standard deviations. The Chi-square test (*χ* ^2^) and Student's *t*-test were used for analysis. For all tests, confidence interval and *P* value were set at 95% and ≤0.05, respectively.

## 3. Results

The present study was carried out on a total of 2000 children (974 (48.7%) males and 1026 (51.3%) females) in the age group of 3 to 5 years.  Table 1 shows the prevalence of dental caries (48%) in preschool children of Kanpur district. The mean dmft was 2.03 ± 2.99. Boys (57%) were affected more than girls (43%) which was found to be statistically significant (*P* < 0.05). The mean dmft of 1.58 ± 0.57 and 1.52 ± 0.59 among boys and girls, respectively, was almost equal and on applying *t*-test, results were found to be nonsignificant (*P* > 0.05).  Table 2 shows the results of intra analysis of dmft components. Out of 4055 dmf teeth, 3968 (97.9%) were decayed, 33 (0.8%) were missing, and 54 (1.3%) were filled.

Table 3 describes the relationship between the proportion of children with visible caries experience and mothers' practices regarding feeding and oral hygiene practices. The majority (60%) of the children were breast fed for 5–10 times. The prevalence of dental caries showed an inverse relationship with the frequency of breast feeding, but the difference was not statistically significant (*P* > 0.05). The duration of breast feeding was 1 to 1.6 years among 46.7% of the study population. The prevalence of caries escalated from 32% to 60.2% along with the increase in duration of breast feeding which was statistically significant (*P* < 0.05). More than half of the study population (52.3%) were not used to the practice of bottle feeding. The prevalence of caries was higher in the population in whom introduction of bottle feeding was delayed to around 2 years which was statistically highly significant (*P* < 0.001).

Of the 954 children with bottle feeding, prolonged duration beyond 2 years was observed among higher proportions (32.2%). Caries experience consistently increased from 30.8% to 75.9% in relation to the duration of bottle feeding which was statistically highly significant (*P* < 0.001). Similarly, significant results were obtained for the quantity of sugar added in the milk. Caries prevalence was 100% among those who had 2-3 tsp of sugar. Corresponding to the relationship between dental caries prevalence and frequency of child falling asleep with the bottle, of the 954 children with bottle feed 33 were falling asleep with bottle feed throughout night. Caries prevalence was 97% in this group which was statistically significant (*P* < 0.05). In children who never fell asleep with bottle feed (500), the prevalence was 51.6%.

Those brushing once a day (61.7%) had a caries prevalence of 47%. However, the relationship between dental caries prevalence and frequency of tooth brushing did not differ significantly (*P* > 0.05). The method of toothbrushing was nothing specific in a minor group of population (8%). Higher proportion of children (54%) practiced both horizontal and vertical methods of brushing and had a caries prevalence of 46.9%. Among those who followed roll on technique (13.3%), the caries prevalence was less (37.5%). The difference observed was statistically nonsignificant (*P* > 0.05). Greater percentage of children had mothers' assistance in cleaning their teeth every day (90.7%). The caries prevalence was low (46%) in this group of population in comparison to other children and the difference is not significant (*P* > 0.05).

## 4. Discussion

Despite widespread preventive measures, dental caries continues to be a veritable scourage for mankind even today [13]. An analysis of caries experience in recent years, especially in developing countries, demonstrates that a significant proportion of infants and preschoolers are still affected by the disease with a strong polarization [14]. The best way of motivating preschoolers towards good oralhealth is through the parents. Children's preventive practices tend to be controlled by their parents' actions and attitudes. For the implementation of preventive attitudes in a given population, the knowledge about the existing standards of health and existing practices and attitudes of that particular population is essential. Since the mother is usually the primary adult carer for the child, it is precisely for this reason that this study was aimed at assessing the practices regarding feeding and oral hygiene habits of Kanpur pre-school children and to evaluate caries experience of their children.

The prevalence of ECC in our study population was 48%. A similar trend of caries prevalence was reported by Dini et al. (2000) [15]. Lower caries prevalence ranging from 8.9 to 36% was reported by Vignarajah and Williams (1992) [16] and Hattab et al. (1999) [17]. Trend of higher caries prevalence ranging from 52% to 98% was reported by Hugoson et al. (2000) [18]. Untreated decayed teeth dominated the dmft score (2.03 ± 2.99) among the children in this study which indicates a high rate of unmet treatment needs. This may be because of the lack of oral health awareness in parents, unhealthy feeding habits and oral hygiene practices, high cost of dental treatment, and limited accessibility and availability of dental services. Similar trends were observed by Chawla et al. (2000) [19] and Hugoson et al. (2000) [18] in their studies. It corroborates Uttar Pradesh National Oral Health Survey Reports, which describe high caries prevalence rates in the primary dentition of under 5-year-old children [20]. A similar trend of caries was reported in Dharwad in 1999 [21], Ahmedabad in 2006 [22], and in eastern states of India like urban areas of Sikkim [23].

In the present study, males were more affected (57%) than females (43%). Similar trends were reported by Al Hosani and Rugg-Gunn (1998) [24]. Studies conducted by O'Sullivan et al. (1994) [25] and Hattab et al. (1999) [17] reported no sex difference in caries prevalence. However, the mean dmft was almost equal and statistically insignificant (*P* > 0.05). Thus, it is inferred that difference in sex is not a risk factor. This may be because at this early age dietary and oral hygiene practices related to dental caries are mostly controlled by parents/caregivers and equally shared.

Caries experience was high in those children who were breast fed <5 times a day. This percentage decreased when the frequency of breast feeding was more than 10 times, but the difference was not statistically significant (*P* > 0.05). Roberts et al. (1994) [26] in their study on 1–4-year-old children found that frequency of feeding was not related to caries prevalence. Children who were breast fed for longer duration demonstrated a higher prevalence of caries which was statistically significant (*P* < 0.05). Results were similar to the findings of Hattab et al. (1999) [17] and Dini et al. (2000) [15].

The prevalence showed an increasing trend for caries in relation to the age at which bottle feeding was introduced. Similar findings were observed by Vignarajah and Williams (1992) [16] in their study. An increasing trend of caries prevalence was observed in relation to the duration of bottle feeding which was similar to the findings reported by Febres (1997) [27]. However, consistent with the literature on dietary practices was the finding of a significantly greater proportion of children with caries among those who added 2-3 tsp of sugar to milk a day. A highly significant association between frequency of sugar consumption and caries in children was seen. Similar findings reported by King [28] and Hattab et al. (1999) [17] showed that children acquire their dietary and oral hygiene habits from parents. Sugars are not only used as a food, but are also given for other reasons, such as taste, as a pacifier and a means of showing love and affection. Higher prevalence of caries was observed in those who fell asleep with bottle throughout night. Febres (1997) [27] in their study found that incidence of caries was higher in children who slept with bottle than those who did not.

Promoting tooth brushing in preschool children is of great relevance because this is a way to favor dental health by maintaining clean teeth. Though the caries prevalence was lower among those children brushing their teeth thrice daily, the difference in the observations obtained was not statistically significant (*P* > 0.05). The findings of the present study were similar to the findings of Bjarnason et al. (1995) [29] and Kuriakose and Joseph (1999) [30]. This could be due to other factors like type and amount of toothpaste and also the method of brushing used. Higher caries prevalence was observed among those practicing horizontal method of brushing in comparison with those following roll on technique; however, it was statistically nonsignificant (*P* > 0.05). Young children can be advised for roll on technique keeping in view the easy practicability and dexterity of brushing. Similar findings were reported by Wei and Hyman (1982) [31].

Results revealed a least prevalence of caries among children who were assisted by mothers for cleaning their teeth. This suggests that preschool children should always be assisted during brushing. Bjarnason et al. (1995) [29] and Kuriakose and Joseph (1999) [30] reported similar findings in regard to caries prevalence and mother assistance in cleaning teeth.

Even though the multifactorial aspect of the etiology of ECC is now well established, the question of why its risk of occurrence is highest among some group is unanswered. When considering possible explanations, dietary habits and sweetened food intake frequency may be likely contributing factors.

### 4.1. Limitations of the Study

The possibility of selection bias must be considered in this study. Not all children in this age group attend preschools or daycare facilities and their behavioural characteristics may have differed from those children sampled through preschool enrolment. Furthermore, being cross-sectional in design, the context and temporal effect of the identified risk factors may not be clear.

## 5. Conclusions

The prevalence of ECC in this sample of preschool children in Kanpur suggest, the need for oral health promotion strategies that include more supportive and practical advice for parents and caregivers of preschool children along with improved access to dental care to enable primary prevention and management of ECC. Intervention during early childhood would seem to be a most appropriate action to ensure healthy dental habits throughout life.

## Figures and Tables

**Table 1:** Prevalence of caries in children according to gender.

Gender	Caries free *n* (%)	Caries affected *n* (%)	dmft Mean ± SD
Boys	427 (41.1)	547 (57)	1.58 ± 0.57
Girls	613 (58.9)	413 (43)	1.52 ± 0.59

Total	1040 (52)	960 (48)	2.03 ± 2.99

Test applied: chi-square, (*χ* ^2^ = 50.02, df = 1; *P* < 0.05)

**Table 2:** Intra-analysis of dmft components in the study population.

No. examined	No. affected	No. of teeth examined	Decayed teeth	Missing teeth	Filled teeth	Total dmft
2000	960	39900	3968 (97.9%)	33 (0.8%)	54 (1.3%)	4055 (10.2%)

**Table 3:** Caries prevalence in relation to feeding and oral hygiene practices.

Items		*n* (%)	Caries affected *n* (%)	Caries free *n* (%)	*P* value
Frequency of breast feeding/day	5 times	500 (25)	260 (52)	240 (48)	*χ* ^2^ = 10.95, df = 2, *P* > 0.05
	5–10 times	1200 (60)	580 (48.3)	620 (51.7)	
	>10 times	300 (15)	120 (40)	180 (60)	

Duration of breast feeding/day	<6 months	206 (10.3)	66 (32)	140 (68)	*χ* ^2^ = 55.72, df = 2, *P* < 0.05
	6 months–1 year	473 (23.7)	193 (40.8)	280 (59.2)	
	1–1.5 years	934 (46.7)	468 (50.1)	466 (49.9)	
	1.5 to 2 years	387 (19.3)	233 (60.2)	154 (39.8)	

Age at which bottle feeding was introduced	<6 months	367 (18.4)	160 (43.6)	207 (56.4)	*χ* ^2^ = 180.80, df = 3, *P* < 0.001
	6 months to 1 year	280 (14)	180 (64.3)	100 (35.7)	
	1 to 2 years	306 (15.3)	233 (76.1)	73 (23.9)	
	Not introduced	1046 (52.3)	387 (37)	661 (63)	

Duration of bottle feeding/day	6 months to 1 year	107 (5.4)	33 (30.8)	74 (69.2)	*χ* ^2^ = 79.18, df = 3, *P* < 0.001
	1 to 1.5 years	260 (13)	133 (51.2)	127 (48.8)	
	1.5 to 2 years	280 (14)	173 (61.8)	107 (38.2)	
	More than 2 years	307 (15.4)	233 (75.9)	74 (24.1)	

Quantity of sugar added in the milk	0.5 tsp	433 (21.7)	240 (55.4)	193 (44.5)	*χ* ^2^ = 64.29, df = 3, *P* < 0.05
	1 tsp	260 (13)	173 (66.5)	87 (33.5)	
	2-3 tsp	68 (3.4)	68 (100)	0	
	None	193 (9.7)	93 (48.2)	100 (51.8)	

Frequency of child falling asleep with bottle	Throughout night	33 (1.7)	32 (97)	1 (3)	*χ* ^2^ = 65.11, df = 3, *P* < 0.05
	Only during feeds	121 (6)	100 (83.3)	21 (16.7)	
	Sometimes	300 (15)	187 (62.3)	113 (37.7)	
	Never	500 (25)	253 (50.6)	247 (49.4)	

Frequency of tooth brushing	Once	1233 (61.7)	580 (47)	653 (53)	*χ* ^2^ = 3.04, df = 2, *P* > 0.05
	Twice	747 (37.4)	373 (49.9)	374 (50.1)	
	Thrice	20 (1)	7 (35)	13 (65)	
	Rarely	0	0	0	

Method of tooth brushing	Nothing specific	160 (8)	93 (58.2)	67 (41.8)	*χ* ^2^ = 35.12, df = 4, *P* > 0.05
	Horizontal	267 (13.5)	160 (59.9)	107 (40.1)	
	Vertical	226 (11.4)	100 (44.1)	126 (55.9)	
	Both H and V	1080 (54)	507 (46.9)	573 (53.1)	
	Roll	267 (13.3)	100 (37.5)	167 (62.5)	

Mother assistance in cleaning teeth	Everyday	1814 (90.6)	834 (46)	980 (54)	*χ* ^2^ = 41.48, df = 3, *P* > 0.05
	Weekly once	13 (0.6)	13 (100)	0	
	Sometimes	147 (7.4)	100 (68)	47 (32)	
	Never	26 (1.4)	13 (50)	13 (50)	

Test applied: chi-square, *P* ≤ 0.05 statistically significant.
